# Surgical Aspects of Rheumatic Heart Disease

**Published:** 1969-10

**Authors:** T. Holmes Sellors


### RHEUMATIC HEART DISEASE : THE DEVELOPMENT OF SURGICAL TECHNIQUES OVER THE PAST 20 YEARS

**T. Holmes Sellors**

In the treatment of acquired heart disease there has always been conflict between the anatomical or mechanical damage to the valves and the more functional element of the disease of the heart muscle itself. In the present age it is difficult to realize how the actual mechanical factor was ignored for so long, and indeed in my young days we were taught that the dominant factor was the rheumatic involvement of the myocardium. Today the pendulum has swung to the other extreme and only lip service seems to be paid to the muscle factor and all attention is directed towards the effect of the disease on the valves.

To trace back to the beginnings, it was Lauder Brunton who first suggested that a stenosed mitral valve might be divided, but, as we know, it was not until the middle 1920's that the first tentative approaches were made by Cutler and Beck and by Souttar. These few operations were just flashes and indeed Souttar was never offered another patient to operate on. Perhaps unjustly, the surgeons said that the dead hand of medical cardiology rested on the subject for another 25 years. This admittedly is not a completely fair statement because surgery itself was not ready to deal with these conditions properly until the modern techniques of intrathoracic surgery had been established.

The origin of heart surgery dates back only 30 years, and even less than that because the earlier operations were on the great vessels such as persistent ductus, coarctation, and Blalock's operation. The first intracardiac operation was on the pulmonary valve in 1947 and it was about this time that we were learning to control bleeding from the heart. In other words, an incision could be made into any of the chambers of the heart without risk of too great a haemorrhage. It was then only a matter of a year or so before surgeons in many parts of the world were studying the possibilities of dealing with mitral stenosis.

This was, after all, a simple concept in theory. If the valve could be disrupted along the pathological lines of fusion, the obstruction to the circulation would be relieved. It remains a debatable point, and one that it is not really necessary to pursue too far, as to who did the first successful mitral valvotomy. In North America one recalls the names of Smithy, Harken, and Bailey, and in our own country Brock, who had successes at the latter end of 1949. It is one of the interesting and extraordinary features of development in surgery that, once the news of a success is announced, surgeons in all parts of the world are ready to go ahead. This happened with mitral valvotomy, and the trans-atrial operation came into being with a rush; and at the European Congress of Cardiology eighteen months to two years later Bedford and I were able to record 50 mitral valvotomies without a death. This was the start of intracardiac surgery. It had the disadvantage of being a blind operation, and only gauged by touch, but this operation has been performed many thousands of times with a mortality of probably about 5%, and good results in over two-thirds of the patients. The risk of recurrence could only be gauged by the passage of time.

Blind valvotomy is an obviously crude procedure, whether it is done with finger, knife, or dilator, but it can be remarkably efficient and one of the factors that contributed greatly to the operation's success was a better understanding of the anatomy. If one looks at the early diagrams of the valve and then at the more recent concepts, the change can readily be appreciated. The operation underwent considerable improvement when the trans-atrial approach was supplemented by trans-ventricular instrumentation of which Logan was one of the earliest advocates.

During this stage of development, attention was given to aortic stenosis but it was found that the surgeon was called upon to deal with a much more severely distorted and calcified valve than in the case of the mitral. Indeed at one demonstration a cardiologist showed a number of specimens in which it was impossible to divide the valve commissures even with bone forceps. On the other hand, it was perfectly possible in some cases to explore the aortic valve with a bougie and if necessary to dilate or disrupt part of the valve with a dilator. Brock and Somerville produced a remarkable series in which the mortality was much lower than in the experience of most of us. The results were reasonably satisfactory and Bailey made out a case for approaching the valve from above, using a sleeve or pouch sewn onto the aorta through which a finger and instrument could be introduced. This was enthusiastically received for a short period, but become unpopular when it was realized how badly damaged the aortic valve usually was and how little could be achieved.

To turn to the next pressing problem, that of mitral regurgitation, this was approached in the early days by a whole series of operations using stitches, pericardial slings, or plication, which produced very little benefit. Things had to wait for the development of open heart surgery whereby both mitral and aortic valves could be seen and their repair carried out.

This brings us almost up to the present age of open heart surgery under perfusion. The aortic valve was first treated by division of commissures, excision of calcium, lengthening of the cusps, and a host of ingenious procedures which have almost entirely given place to excision and valve replacement. In the case of the mitral valve the supply of the pure mitral stenoses that we used to encounter and operate on at the rate of four or five a week has almost dried up. Most of the valve damage now is a mixture of stenosis and regurgitation or else pure regurgitation. Again, as in the case of aortic valve, operations aiming at advancing the cusps or at special types of suture and plication seem to be giving place to the more radical excision and replacement. Some surgeons indeed are advocating treating even pure mitral stenosis by open operation, but in view of the good records of the simpler form of treatment, I find this hard to justify.

I have used the term replacement rather vaguely, but behind this term lies a whole mass of experimental work that is aimed at producing an efficient valve that the heart will tolerate. Ideally homografts meet most of the requirements. They are accepted and incorporated with only few examples of rejection, but whereas the aortic valve only offers a few problems the mitral valve homograft has proved much more difficult, and indeed replacement with a reversed aortic homograft or heterograft has met with greater favour.

If human tissue is not available or suitable, then a prosthesis has to be used, and in spite of many types of valve designed, the ball valve with its cage seems to be the most popular. It is, of course, mechanically unsound because it is rigid and made of foreign material, and no-one can pretend that the flow through such a valve can approximate the normal, but sufficient of the Starr-Edwards pattern have been consigned to human aortas to have proved their worth. Flap valves, hinge valves, all sorts of valves, have been produced; but if one remembers that each valve beats nearly a million times a week a considerable degree of stress is encountered.

To continue with the present position of aortic valve surgery, the technique seems fairly well established in that all operations are carried out under perfusion with or without some degree of cooling, and after the aorta has been cross-clamped it is incised transversely or obliquely and almost immediately both coronary arteries are cannulated and perfused. The valve is then inspected, and the degree of stenosis or regurgitation does not make very much difference, because with few exceptions a repair operation such as division of commissures is not likely to be of value, and certainly will be of little use if the valve is fibrosed and calcified. The damaged valve is excised and a decision is made as to the use of a homograft or prosthesis. Sutures will then be placed below the coronary ostia in the region of the excision, and after three equally spaced guy stitches have been inserted about seven or eight other additional stitches will be placed in each of the three sections between these guys. These stitches are sewn into the homograft or prosthesis which is then slid down and secured in its new bed, and the valve is tripped for exclusion of air at a later stage. In the case of the homograft the three commissural buttresses have to be brought up to hold the cusps in place, and this operation takes longer and requires more expertise than inserting a prosthesis. Finally, the aorta is closed, all air is removed, and as the last stitches are inserted the coronary cannulae are removed and perfusion is slowly stopped. The heart may take over quite well, but when there is gross enlargement of the left ventricular muscle to an extent that the actual cavity is reduced in size, the procedure may not be straightforward and careful watch to combat any onset of ventricular fibrillation must be made.

A recent elaboration of aortic valve replacement was introduced by D. Ross, who has on occasions excised the patient's pulmonary valve and used it as an autograft and then replaced the pulmonary valve with a homograft or prosthesis. In this way a more suitable valve is placed in the area of high stress, and replacement of the pulmonary valve does not seem to cause much difficulty.

Initially, as with all forms of surgery, the original results of valve replacement left much to be desired, but in units where the experience with this operation is considerable mortality figures in the region of 10% or 12% can now be expected; less in experienced hands.

The position as regards mitral valve surgery has already been indicated. The immense supply of pure mitral stenosis has come almost to a halt, and most patients requiring surgery show a degree of regurgitation that makes it imperative to explore the valve by open techniques and this particularly applies in the presence of calcification. The approach to the valve can either be through a vertical incision, followed by opening of the *right* atrium, and then dividing the septum completely to expose the mitral valve itself, or through the left chest for a left atrial approach. Occasionally a local repair can be achieved, but with calcification and much distortion replacement is the probable answer. The ball valve and cage type has certain disadvantages if the cavity of the ventricle is small, an objection that does not apply to the flap valve type.

Homografts of the mitral valve itself have proved difficult to insert satisfactorily owing to the problem of fixing the chordae. Aortic valves, reversed and possibly sutured onto a ring, have been used with increasing success. Some surgeons prefer a left sided approach if there has been no previous operation and find that access to the valve through the left atrium is straightforward.

The complications of mitral valve surgery, particularly in the presence of fibrillation, are essentially those of emboli. Small pieces of clot tend to go to the brain, producing varying degrees of damage, and larger clots may impact on the aortic bifurcation and require removal as a matter of urgency.

When it comes to the late complications, if a prosthesis has been used the risks of embolism have always to be recognized, and anticoagulants will be the lot of most of these patients in the hope of preventing this disaster. Valves can work loose from their sutures and produce leakage, but the benefit produced by a mechanically efficient new valve may well be life-saving.

Recurrence of mitral stenosis after valvotomy is an acknowledged complication possibly amounting to 7 to 10% of all cases every five years, but there is a great deal of muddled thinking as to what is meant by the term re-stenosis. I usually regard this term as including four different conditions:—

1) Recurrence of rheumatic endocarditis can obviously lead to the commissures sticking together again, and this, though rare, has to be recognized.
2) The operation of valvotomy may have been inadequate and under this heading I would include the first dozen or so in any surgeon's experience before he has gained sufficient confidence to perform an adequate operation. It also includes cases in which one commissure, because of fibrosis or calcium, has been deliberately left alone.
3) The case in which at the second operation the opening in the mitral valve measures precisely the same as at the time of the original valvotomy. Here I am convinced that there is a relative re-stenosis produced by myocardial weakness.
4) Actual recicatrisation of the orifice, which in my experience is rare, though it undoubtedly can occur in a proportion of cases.

The term re-stenosis should at the present time be less common because in the early days many patients treated by closed valvotomy were (in present thinking) unsuitable for closed surgery and would now be treated by open exploration with repair or replacement.

A final word may be said about multi-valve disease. In the early days, to avoid unnecessary risks, the more important or more grossly damaged valve was usually attempted first, though on occasions blind mitral and aortic valvotomy was done with a dilator in one operation. The present policy appears to be that unless you can render all valves efficient at the same time, the chances of doing a successful staged operation are small. No one can admit that double or triple valve operations are as safe as a single one, partly because of the time under perfusion, and partly because of the actual trauma inflicted on various parts of the heart, which itself is already seriously damaged; but if the patient is in severe heart failure and would not be expected to live more than a very short time he may have to be accepted for operation even at considerable risk.

My concluding remarks refer simply to matters of observation. No one has ever explained satisfactorily to me why the mitral and aortic valves in the high pressure circuit bear the brunt of rheumatic disease and why the low pressured tricuspid and pulmonary valves escape; and I should be interested to know when the pendulum will swing back from concentration on valve efficiency to an assessment of the damage that has occurred to the actual muscle.

